# Covalent recruitment of polymers and nanoparticles onto glycan-engineered cells enhances gene delivery during short exposure†

**DOI:** 10.1039/d4sc03666b

**Published:** 2024-09-09

**Authors:** Qiao Tang, Ruben M. F. Tomás, Matthew I. Gibson

**Affiliations:** a Department of Chemistry, University of Warwick Gibbet Hill Road Coventry CV4 7AL UK matt.gibson@manchester.ac.uk; b Division of Biomedical Sciences, Warwick Medical School, University of Warwick Gibbet Hill Road Coventry CV4 7AL UK; c Cryologyx Ltd 71-75 Shelton Street London WC2H 9JQ UK; d Department of Chemistry, University of Manchester Oxford Road Manchester M13 9PL UK; e Manchester Institute of Biotechnology 131 Princess Street Manchester M1 7DN UK

## Abstract

Non-viral gene delivery with cationic polymers/nanoparticles relies on iterative optimization of the carrier to achieve delivery. Here we demonstrate, instead, that precision engineering of cell surfaces to covalently capture a polyplex accelerates gene delivery within just 10 min of exposure. Azides were installed into cell-surface sialic acids, which enabled the rapid and selective recruitment of cyclooctyne-functional polyplexes, leading to increased delivery of fluorescent cargo, and also increased plasmid expression and siRNA knockdown. Covalent delivery enhancement was also shown for a polymer-coated nanoparticle delivery system. This validates using cellular metabolic engineering (or other synthetic biology) tools to overcome payload delivery challenges.

## Abstract 2

Engineering cell surface glycans with azides promotes the capture of polyplexes to increase genetic material delivery into cells.

## Graphical abstract

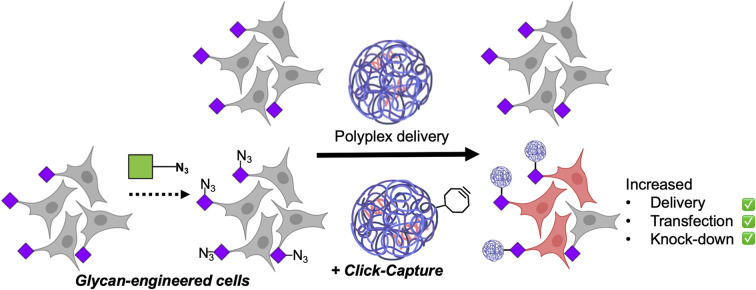

## Introduction

Viral-vector gene delivery has reached the clinic but there remain challenges including high costs and immunogenicity concerns.^1,2^ Synthetic delivery vectors are widely explored, including cationic lipids (as used in mRNA vaccines), and polycations to condense genomic materials into polyplex delivery vehicles. Polymeric delivery vehicles face the challenge that gene delivery efficacy and cytotoxicity are often antagonistic design principles, along with requiring optimization for intracellular trafficking and release. 100's of different polymeric delivery vehicles has been reported, but identifying an optimal polymer structure remains challenging.^3,4^ Multiple structural parameters determine transfection and release, including hydrophobicity, monomer composition^5^ and architecture.^6^ Many methods have been applied to find the optimal polymer structure such as high throughput synthesis/screening and artificial intelligence-driven prediction, deliver nucleic acids (*e.g.* pDNA,^7^ siRNA, siRNA, saRNA, mRNA^8^) and similar challenges/approaches exist for intracellular protein delivery.^9^

The above examples highlight that the current design paradigm is to ‘*engineer the carrier*’ to maximize delivery. An alternative approach to this problem is to ‘*engineer the target cells*’ to make them more receptive to delivery or to capture the vehicle. For example, cell-surface thiols can react with maleimide-nanoparticles, and lipid insertion can anchor polymers,^10^ but are non-selective.^11^ If one considers the external surface of all mammalian cells, the main interface is the dynamic, glycosylated layer: the glycocalyx. The glycocalyx performs roles from cell–cell communication, as pathogen adhesion sites and to drive tumour-cell growth. Metabolic oligosaccharide engineering (MOE) enables chemical editing of the periphery of cell surfaces by the installation of unnatural sugars into the glycocalyx.^12^ Ac_4_ManNAz (*N*-azidoacetylmannosamine-tetraacylated) can diffuse into cells where it is deacetylated and subsequently enters the sialic acid biosynthesis pathway, resulting in azide integration into sialic acids at the terminus of cell-surface glycans. Addition of a complementary functionality (*e.g.* alkynes) allows for cell-surface labelling and has been achieved using a range of different glycans and reaction partners. Polymer nanoparticles can be directed to glyco-engineered cells for radiotherapy,^13^ chemotherapy^14,15^ and to target chemo-therapeutic membrane disrupting polymers to the cell surface.^16^ Selectivity with glyco-engineered cells can be achieved by exploiting metabolic gradients,^17^ engineering cellular pathways,^18^ chemical caging of unnatural glycans^19^ and design of cell-specific glycans for labelling.^20,21^

In the context of gene delivery, glycans are an appealing target for cell-surface editing:^22,23^ MOE results in homogenous labelling at the cells exterior, unlike many gene delivery vectors which do not transfect all cells in a given population.^24^ Glycans are also recycled from the cell surface *via* endosomes and hence offer a pathway to the required intracellular locations.

Herein, we demonstrate that azido glycan engineered cells can capture complementary alkyne-polyplexes leading to increased gene delivery during short (10 minute) exposures, and is also shown to function with a nanoparticle formulation. This significantly outperforms non-edited cells, and also commercial transfection agents, allowing rapid and homogeneous delivery of plasmids and siRNA into a panel of cell lines. This shows that glycan (or other) cell editing tools could be deployed to enhance the rate and homogeneity of gene delivery to improve selectivity and efficiency of delivery.

**Fig. 1:**
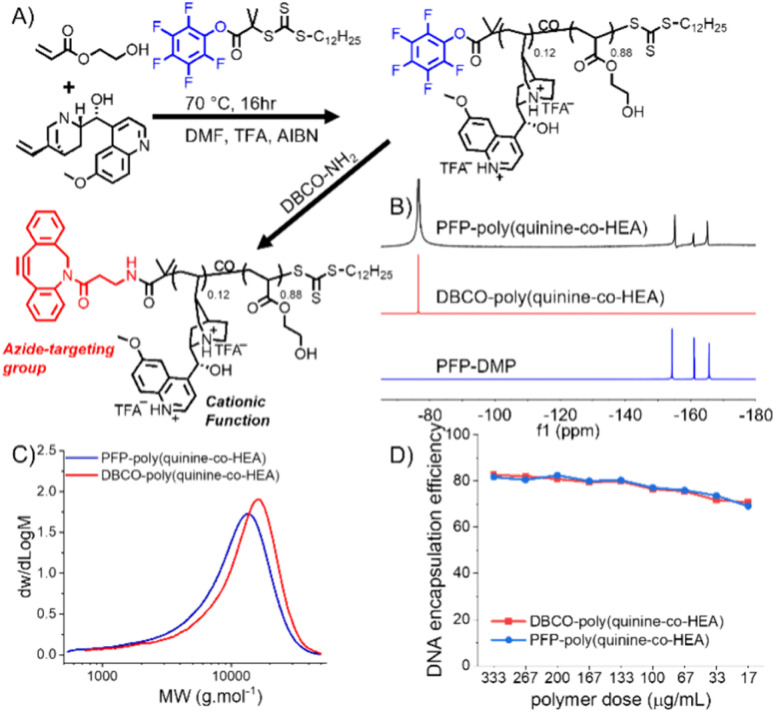
Covalent delivery concept and polymer synthesis. (A) Synthesis of DBCO-poly(quinine-*co*-HEA) and poly(quinine-*co*-HEA); (B) ^19^F-NMR, (C) DMF-SEC molecular weight distributions and (D) dye exclusion assay for pDNA encapsulation efficiency.

In our initial experiments dibenzylcyclooctyene (DBCO for strain promoted click)-functionalized poly(dimethylamino ethyl methacrylate), PDMEMA, was synthesized (as a model cationic polymer) and tested for click-targeted gene delivery. A549 cells were labelled using Ac_4_ManNAz to introduce azides into cell-surface sialic acids. During this screening the delivery was inefficient. Therefore, an alternative cationic copolymer based on quinine/2-hydroxyethyl acrylate (HEA), first reported by Reineke and coworkers, was identified.^25^ HEA and quinine were copolymerized with 2-(dodecylthiocarbonothioylthio)-2-methylpropionic acid pentafluorophenyl ester (PFP-DMP) to install a terminal PFP group (for DBCO-NH_2_ conjugation) (Fig. 1A and S1†).

^19^F NMR showed the PFP group was lost during polymerization, which was solved by addition of TFA (trifluoroacetic acid) to mask the pyridine nucleophile. Quinine incorporation into the polymer was reduced when TFA was used, and hence a feed ratio of quinine : HEA of 60 : 40 was needed to produce a polymer with 12 mol% quinine (above the 10% threshold required for efficient gene delivery),^25^ and ^19^F NMR confirmed PFP was retained (Tables S1 and S2†) (Fig. 1B, C and S3†). No further attempts were made to optimize this synthesis as our primary aim was the click-delivery (below). With PFP-poly(quinine-*co*-HEA) to hand, DBCO-NH_2_ was installed by PFP displacement to generate the click-targeted polycationic vector. Poly(quinine-*co*-HEA) without DBCO (poly(quinine-*co*-HEA)) was used as negative control throughout.

Polyplex formation using a GFP (green fluorescent protein)-encoding plasmid was confirmed using gel electrophoresis (Fig. S4†). A dye exclusion assay (PicoGreen) quantified that both poly(quinine-*co*-HEA) and DBCO-poly(quinine-*co*-HEA) gave >90% DNA condensation efficiency at all N : P's (Fig. 1D). A pro-fluorescent azido-dye (3-azido-7-hydroxycoumarin) was used to show that the DBCO groups were equally available in the polyplex, compared to the polymer alone, indicating the DBCO groups are available for clicking onto cell surface azides (Fig. S5†). Dynamic light scattering showed the polyplexes were ∼200 nm with a net positive charge, which increased with N : P ratio (Table S3†). These data show that the polymers with or without DBCO have similar properties.

**Fig. 2:**
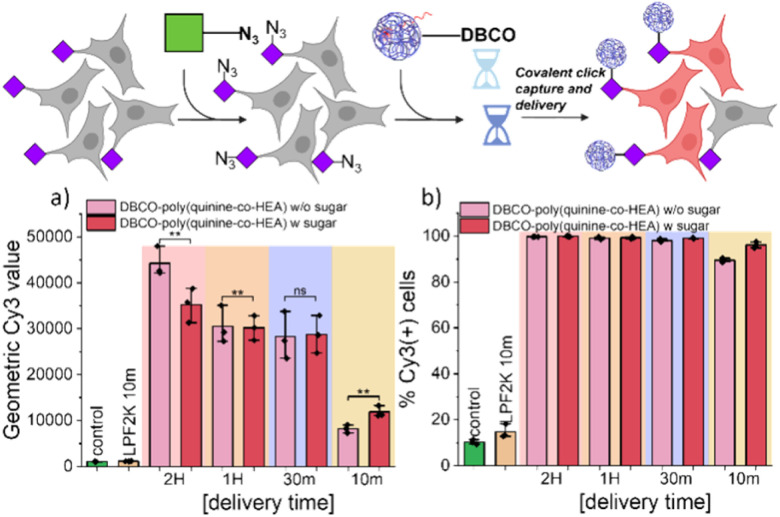
Time dependent delivery screening of siRNA-Cy3 by DBCO-poly(quinine-*co*-HEA) into A549 cells with or without azido-sialic acid installation. Fluorescence intensity of cells was analysed immediately after delivery. (a) Geometric mean Cy3 value of cells; (b) Cy3 positive cells under different conditions. w-sugar = Ac_4_ManAz labelled cells, w/o means without. *N* = 3 (paired sample *t* test; ns: *p* ≥ 0.05, *p ≤ 0.05, **p ≤ 0.01, ***p ≤ 0.001).

To evaluate covalent ‘click’ targeting, a Cy3 (fluorophore) labelled siRNA (siCy3) was first used. This probe allows delivery to be probed (by fluorescence measurements) independently and faster than functional gene expression/knock down assays (explored later). A549 cells were incubated with 50 μM Ac_4_ManNAz for 72 h to install azido-sialic acids on the cell surface (validated using DBCO-dyes, Fig. S6 and S7†). Cells were exposed to polyplexes containing siCy3 for 10–120 min, washed with DPBS and immediately trypsinized for flow cytometry, ensuring whole population analysis. Fig. 2A shows the average fluorescence for each set of conditions, per time point and controls of untreated cells (for the baseline).

Covalent recruitment of DBCO-poly(quinine-*co*-HEA)/siCy3 polyplexes showed maximum delivery enhancement (compared to controls) during very short exposure times (10 min) which was more pronounced than for non-DBCO polyplexes (Fig. S8†). This shows the covalent conjugation effects are larger than the small impact of the cell glycan engineering alone. Both geometric mean Cy3 fluorescence intensity and percentage of Cy3 positive cells of DBCO-poly(quinine-*co*-HEA)/siCy3 polyplexes treated A549 cells with Ac_4_ManNAz pretreatment is higher than non-labelled cells. Extended incubation times resulted in less difference (but more overall delivery) between cells with or without the azido-labelled on the cell surface. The negative control of poly(quinine-*co*-HEA) delivered similar amount of siCy3 into A549 cells with and without Ac_4_ManNAz.

These observations reveal that for long polyplex exposure times, the covalent anchoring provides only minor benefits as the electrostatic attraction can reach a presumed equilibrium/saturation. Whereas for short incubation times the click-capture renders polyplex/cell engagements irreversible and hence increases the residence time/extent. Comparison with Lipofectamine 2000 resulted in only 20% cell transfection under the same delivery time (Fig. 2b) showing our delivery strategy is superior under these short-term incubation conditions and that the use of a covalent ‘anchor’ on the cell surface can enhance gene delivery.

**Fig. 3:**
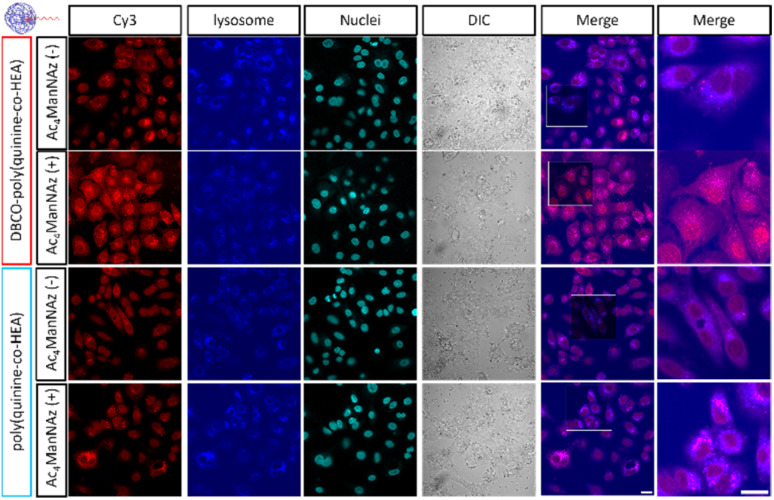
Confocal images of siRNA-Cy3 10 min-delivery by DBCO-poly(quinine-*co*-HEA) and poly(quinine-*co*-HEA) on A549 cells with or without 72 h 50 μM Ac_4_ManNAz pre-treatment. Red: siRNA-Cy3; blue: lysosome; cyan blue: nuclei; grey: bright field. Scale bar: 20 μm (confocal images are applied with the same brightness and contrast increasement and original images are in Fig. S9†).

Confocal microscopy of cells with or without Ac_4_ManNAz pre-treatment showed similar fluorescence at 12 h post-delivery when using poly(quinine-*co*-HEA)/siCy3, Fig. 3. However, DBCO-poly(quinine-*co*-HEA)/siCy3 delivered more siCy3 into cells with Ac_4_ManNAz pre-treatment than cells without any. There was also colocalization with lysosomes confirming the polyplex delivery pathway is similar to non click-targeting (Fig. S9† for Pearson coefficients and Fig. S10† for quantitative MFI), *via* endocytosis.

**Fig. 4:**
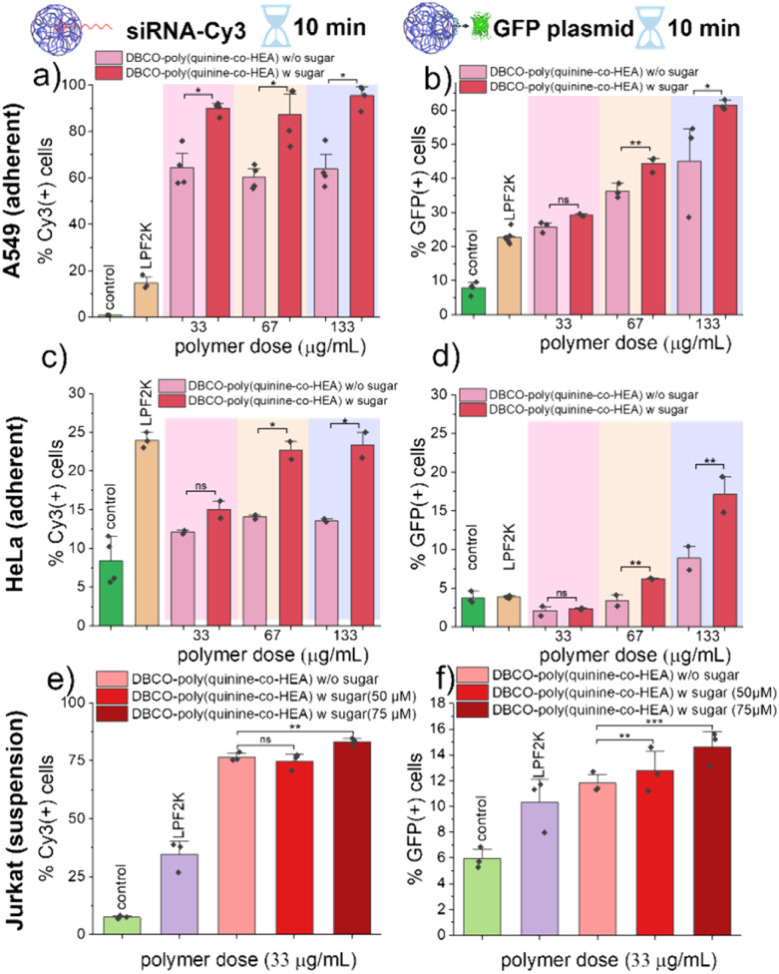
siRNA-Cy3 and GFP plasmid delivery by DBCO-poly(quinine-*co*-HEA) into three cell lines. Cells were analysed on flow cytometer immediately after delivery for siCy3 delivery and 48 h-post-delivery for GFP plasmid delivery. A549 cells: (a) siCy3 and (b) GFP plasmid; HeLa cells: (c) siCy3 and (d) GFP plasmid; Jurkat cells: (e) siCy3 and (f) GFP plasmid. *N* = 3 (paired sample *t* test; ns: *p* ≥ 0.05, *p ≤ 0.05, **p ≤ 0.01, ***p ≤ 0.001).

The above screening confirmed that covalent targeting can increase gene delivery but the differences observed under those conditions were smaller. Therefore, optimization was undertaken to maximize delivery. The polymer dose was varied from 0 to 200 μg mL^−1^ and cytotoxicity evaluated (Fig. S11†). 75% of Ac_4_ManNAz pretreated A549 cells were viable up to 200 μg mL^−1^ of polymer, with DBCO being slightly more toxic in azido-cells due to covalent recruitment of the cationic polymers (more cell membrane damage), as would be expected.^16^ With polymer dose increasing, overall uptake efficiency of DBCO-poly(quinine-*co*-HEA)/siCy3 into cells with and without Ac_4_ManNAz pre-treatment resulted in more delivery (enhancement was increased up to 40%) due to the covalent clicking approach, Fig. 4a.

To validate rapid transfection (not just delivery), a GFP-encoding plasmid was next delivered. As shown in Fig. 4b, DBCO-poly(quinine-*co*-HEA)/GFP plasmid showed higher transfection efficacy (more fluorescence) with Ac_4_ManNAz pre-treatment, compared to cells without. This confirms that the covalent anchoring onto cell surface glycans does not prevent successful genetic material transfection. Controls of poly(quinine-*co*-HEA) showed similar transfection efficacy on both azido-cells and bare cells under low dose but showed higher efficacy on azido-cells with higher polymer dose (Fig. S12b†). The reason for this is not clear as it was not shown in the total delivery data (Fig. 4a), but maybe due to the high extent of delivery at higher concentrations meaning the covalent conjugation has less benefit.

To further validate this method, additional cell lines were screened for both delivery and transfection: adherent HeLa and suspension Jurkat cells, Fig. 4c–f. HeLa cells did not show a statistically significant increase in siCy3 delivery and GFP expression with the Az-labelled cells using DBCO polymer. Jurkat cells showed some increases with this strategy. Polymer cytotoxicity was evaluated on above cells and showed around 75% viability at tested concentration (Fig. S13 and S14†). In all cases performance matched or exceeded lipofectamine 2000 delivery. We note that even though lipofectamine 2000 showed highest percentage of Cy3 positive cells in Fig. 4c, its geometric Cy3 value is lower than DBCO-poly(quinine-*co*-HEA) on Az-labelled cells, data shown in Fig. S15.† Gene delivery into suspension cells (Jurkats) is known to be more challenging than adherent,^26,27^ with factors including glycan-recycling rate contributing.^28^ Whilst outside of the scope of this work, there are many different glycan labels to hijack biosynthetic pathways which could be fine-tuned per cell type,^20^ to be explored in the future.

**Fig. 5:**
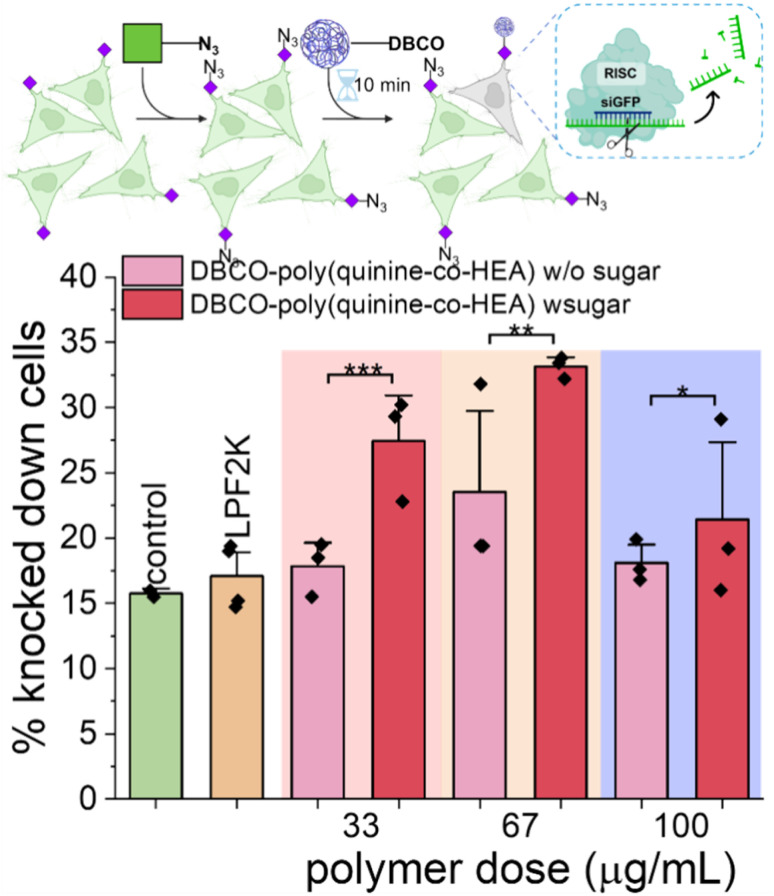
Dose dependency of siGFP delivery by DBCO-poly(quinine-*co*-HEA) on HeLa-GFP cells with or without 72 h 50 μM Ac_4_ManNAz pre-treatment. *N* = 3 (paired sample *t* test; ns: *p* ≥ 0.05, *p ≤ 0.05, **p ≤ 0.01, ***p ≤ 0.001).

To further demonstrate the versatility of this click-targeting concept, gene knocking down was tested using siRNA (rather than the labelled siRNA used earlier). HeLa cells stably expressing GFP were targeted by DBCO-poly(quinine-*co*-HEA)/siGFP, with success determined by a decrease (*i.e.* silencing) in GFP expression. As shown in Fig. 5, DBCO-poly(quinine-*co*-HEA)/siGFP (67 μg mL^−1^) knocked down 35% azido-HeLa-GFP cells and 22% unlabeled HeLa-GFP cells, respectively, again showing that covalent targeting enhances activity with our rapid delivery times. Lipofectamine 2000 reduced GFP by only 15% and poly(quinine-*co*-HEA)/siGFP reduced GFP by 25% protein loss on both azido-HeLa-GFP and bare HeLa-GFP cells (Fig. S16†). Knocking-down efficiency increased with polymer dose increasing until cytotoxicity of the polycations became too significant (Fig. S17†). This data clearly shows that metabolic labelling, and covalent capture approach increases the rate of gene delivery, and that the unique mechanism does not interfere with successful gene expression nor knock-down.

**Fig. 6:**
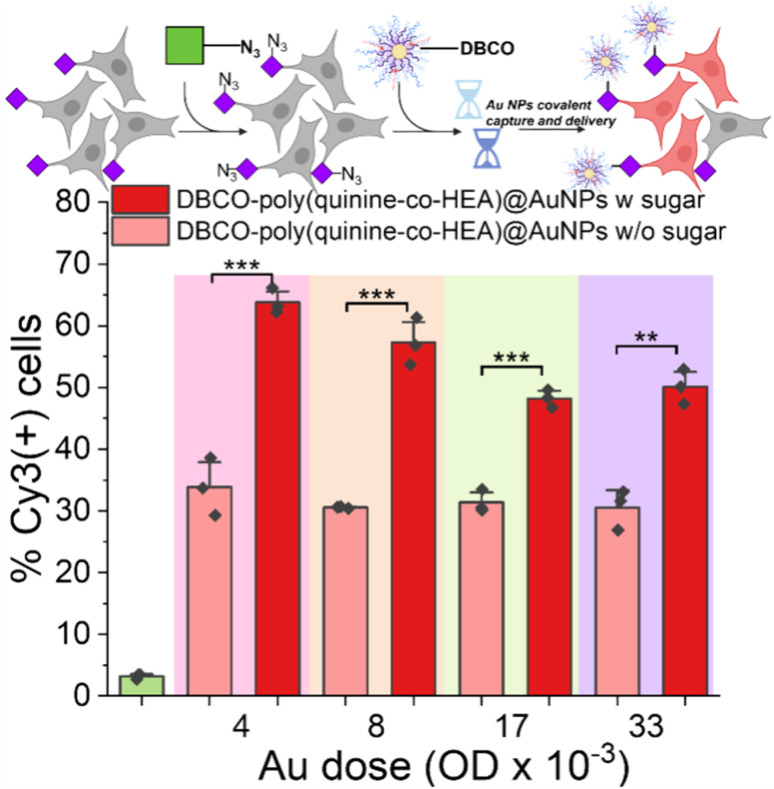
Delivery of siCy3 by Au nanoparticles. 30 nm gold coated with 11-mercaptoundecanoic acid/DBCO-poly(quinine-*co*-HEA)/siCy3/DBCO-poly(quinine-*co*-HEA) (see ESI† for synthesis). A549 cells were used with or without 72 h 50 μM Ac_4_ManNAz pre-treatment. Delivery by poly(quinine-*co*-HEA)@AuNPs was found in Fig. S19.†*N* = 3 (paired sample *t* test; ns: *p* ≥ 0.05, *p ≤ 0.05, **p ≤ 0.01, ***p ≤ 0.001).

As a final demonstration of the versatility and potential broad scope of this delivery enhancement mechanism, gold nanoparticle/polymer hybrids were investigated for covalent delivery, guided by reports of enhanced delivery compared to polymers alone.^29,30^ In brief, multilayer AuNPs were assembled from a 40 nm gold particle, first coated with MUA (mercaptoundecanoic acid), then poly(quinine-*co*-HEA) with or without DBCO, followed by siRNA-Cy3, and finally capped with poly(quinine-*co*-HEA) with or without DBCO (Fig. S2†). Polyplex stability was confirmed by dynamic light scattering and the nanoparticle size determined by transmission electron microscopy (TEM) (Fig. S18†). As shown in Fig. 6, delivery to A549 cells was evaluated by flow cytometry (as above) and there was up to two-fold enhancement for the glycan edited cells, compared to unedited. The covalent targeting in this case clearly transformed for efficacy of delivery and with very short exposure times.

It should be noted the extent of delivery using this covalent method, due to the short times, is not as high as in some other optimized systems, but our aim was to show how glycan-edited cells can be used to capture polyplexes and how the approach is applicable to a range of delivery vehicles. This present work also proves the concept that delivery can be achieved without having to overcome receptor–ligand affinity/saturation/selectivity challenges through use of an orthogonal chemical conjugation step. Future work will include optimization, the use of specific glycan labelling to allow cellular specificity to be achieved and experimenting under flow where the enhanced kinetics are expected to bring significant benefits.

## Experimental

Full experimental details are in the electronic ESI.† This includes characterization of all polymers and nanomaterials, alongside additional data.

## Conclusions

To conclude, we have demonstrated that covalent ‘click’ targeting of polymer and nanoparticle polyplexes to cell surfaces bearing azido-glycans leads to significant enhancement in gene delivery, transfection and knock-down, during short exposure times in both polymeric and gold nanoparticle, platforms. Poly(quinine-*co*-hydroxyethyl acrylate) was synthesized by RAFT polymerization to allow installation of an azide-reactive DBCO group into the chain ends, for covalent capture on the cell surface. The polymer was able to deliver siRNA and plasmids into three different cells lines with enhancement after glycan engineering, whilst non-specific recruitment with less enhancement was validated using non-DBCO polyplexes. For adherent cell lines, the covalent targeting increased delivery by up to 40% but the effect was less pronounced on suspension cell lines in line with previous reports on gene delivery to these cells. Whilst not explored here, a range of other (glycan) metabolic labels have been reported, which may allow tuning towards specific cell types. The covalent click-targeting was shown under all conditions to match or exceed Lipofectamine 2000 and was shown in terms of total material delivered, plasmid expression and siRNA knock-down. The largest increases were seen using the gold nanoparticle delivery where two-fold increase in siCy3 were seen. This proof-of-concept study clearly shows that the covalent anchoring approach may overcome limitations in gene delivery and offers an alternative to the current approach of iterative small changes to the polymer carrier, and instead exploits distinct cellular metabolic processes to increase delivery. We anticipate that the enhancements at short incubation times are particularly relevant to *e.g.* under flow or more physiological conditions which will be explored in the future, alongside increasing selectivity though precision metabolic labeling strategies.

## Data availability

Background data is available in the ESI.†

## Author contributions

Conceptualization: MIG. Methodology; RT and QT. Supervision: MIG. Investigation: RT and QT. Writing: MIG and QT.

## Conflicts of interest

There are no conflicts to declare.

## Supplementary Material

SC-OLF-D4SC03666B-s001
