# Supplementary material for: Covalent recruitment of polymers and nanoparticles onto glycan-engineered cells enhances gene delivery during short exposure
Source: Chem Sci. 2024 Sep 9;15(38):15731–6. doi: 10.1039/d4sc03666b (PMC11382546; doi:10.1039/d4sc03666b)
Supplement: SC-015-D4SC03666B-s001 [file SC-015-D4SC03666B-s001.pdf]

Supplementary Information (SI) for Chemical Science.
This journal is © The Royal Society of Chemistry 2024

Electronic Supporting Information For

Covalent Recruitment of Polymers and Nanoparticles onto Glycan-Engineered Cells Enhances Gene Delivery During Short Exposure

Qiao Tang,[1] Ruben M.F. Tomas[2,3] and Matthew I. Gibson[1,2,4,5*]

[1]Department of chemistry, University of Warwick, Gibbet Hill Road, Coventry CV4 7AL, U.K.

[2]Division of Biomedical Sciences, Warwick Medical School, University of Warwick, Gibbet Hill Road, Coventry CV4 7AL, U.K.

[3]Cryologyx Ltd, 71-75 Shelton Street, London, WC2H 9JQ, UK

[4]Department of Chemistry, University of Manchester, Oxford Road, Manchester M13 9PL, UK.

[5]Manchester Institute of Biotechnology, 131 Princess Street, Manchester, M1 7DN UK

*Corresponding Author. Matt.gibson@manchester.ac.uk

General 3
Materials 3
Physical and Analytical Methods 3
Polymer synthesis 5
Polyplexes formation 6
DNA encapsulation efficiency (gel electrophoresis) 6
DNA encapsulation efficiency measurement (dye exclusion assay) 6
Click reaction of azido-dye and DBCO-polymer/polyplexes 6
Gold/polyplexes preparation 7
Plasmid preparation 8
Mammalian cell culture 8
Gene delivery 9
Flow cytometry 10
Confocal microscopy 10
Resazurin assay 11

General

Materials

Quinine, 2-hydroxyethyl acrylate, dibenzocyclooctyne-amine, trifluoroacetic acid, 2-(dodecylthiocarbonothioylthio)-2-methylpropionic acid pentafluorophenyl ester, methanol-d_4 and N-azidoacetylmannosamine-tetraacylated ($Ac_4ManNAz$) were purchased from Sigma-Aldrich. CMV-EGFP plasmid was purchased from SinoBiologics. Invitrogen™ Silencer™ Cy™3-labeled GAPDH siRNA, Invitrogen™ Silencer™ GFP (eGFP) siRNA and E-Gel™ EX Agarose Gels (1%) was purchased from Thermo Scientific. Au nanoparticles (30 nm diameter, OD 1) and 11-mercaptoundecanoic acid were purchased from Sigma Aldrich. 3-azido-7-hydroxycoumarin was purchased from BioServ UK Limited.

Invitrogen™ Qubit™ dsDNA Quantification Assay Kit was purchased from Thermo Scientific. E.Z.N.A.® Endo-Free plasmid mini kit II was purchased from Omega.

Physical and Analytical Methods

NMR spectroscopy. ^{1}H-NMR and ^{19}F-NMR spectra were recorded at 400MHz on a Bruker DPX - 400 spectrometer with methanol-d_6 as the solvent. Chemical shifts of protons are reported as δ in parts per million (ppm) and are referenced to residual non-deuterated solvent.

Size exclusion Chromatography in DMF. Size exclusion chromatography (SEC) analysis was performed on Agilent 390 - LC MDS instrument equipped with differential refractive index (DRI), viscometry (VS), dual angle light scatter (LS) and UV detectors. The system was equipped with 2 x PLgel Mixed D columns (300 x 7.5 mm) and a PLgel 5 µm guard column. The eluent is DMF with 5 mmol NH_4BF_4 additive. Samples were run at 1 ml.min^{-1} at 50 °C. Poly(methyl methacrylate) standards (Agilent EasyVials) were used for calibration between 955,000 - 550 g.mol^{-1}. Analyte samples were filtered through a nylon membrane with 0.22 µm pore size before injection. Respectively, experimental molar mass (Mn, SEC) and dispersity (Đ) values of synthesized polymers were determined by conventional calibration and universal calibration using Agilent GPC/SEC software.

Cell counting. Cells were stained with trypan blue solution at final concentration of 0.1% (w/v)

and then counted under Countess II Automated Cell Counter.

Fluorescent microscopy. Confocal microscopy was performed on Olympus FLUOVIEW FV3000 Confocal Laser Scanning Microscope using 60x oil objective at room temperature under sequential scanning between frames mode.

Flow cytometery. Flow cytometry was performed on BD Accuri™ C6 Plus flow cytometer. GFP signal was recorded under FITC channel (FL-1) and Cy3 signal was recorded under PE channel (FL-2), respectively.

DNA concentration measurement. Plasmid concentration was determined on IMPLEN NanoPhotometer®.

DNA gel electrophoresis. DNA mobility was performed on E-Gel™ Power Snap Electrophoresis System using commercial Invitrogen™ E-Gel™ EX Agarose Gels (1%). Polyplexes of different N/P ratio and bare plasmid at the same plasmid dose of 300 ng were running on gel for 10 min.

Dynamic light scattering. Size of polyplexes with different mass ratios of polymer: plasmid was determined by DLS on Wyatt DLS platereader and size of Au/polyplexes particles were performed on Litesizer™700.

Zeta potential. Zeta potential of polyplexes with different mass ratios of polymer: p and Au/polyplexes particles was performed on a Malvern Zetasizer and Litesizer™700, respectively.

Transmission Electron Microscopy. TEM images and high-resolution transmission electron microscope (HRTEM) images were obtained with an Thermo Fisher Scientific - Talos F200X microscope equipped with an X-FEG electron source. The experiment was performed using an acceleration voltage of 200kV and a beam current of approximately 5 nA. Images were recorded with a Thermo Scientific - CETA 4k x 4k CMOS camera.

Statistical analysis. Pair sample t-test was performed on flow cytometry data of siCy3 and GFP plasmid delivery on cell. In the Figures presenting P value, *** means a P value smaller than/equal to 0.001; ** means a P value smaller than/equal to 0.01; * means a P value smaller than/equal to 0.05; ns means a P value larger than 0.05.

Polymer synthesis

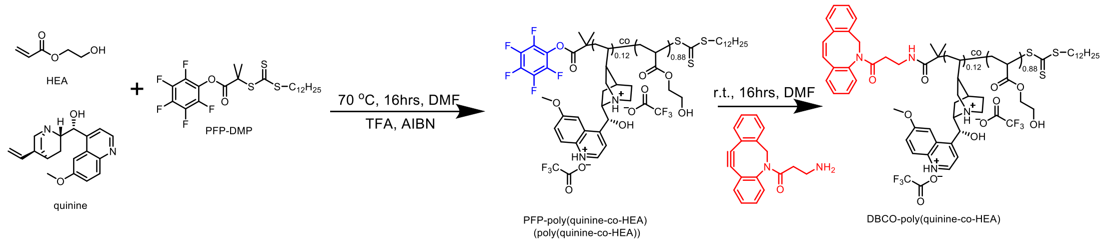

Figure S1. Synthesis route of DBCO-poly(quinine-co-HEA) and poly(quinine-co-HEA).

To target a monomer ratio of 40: 60 and a DP of 100, quinine (7.34 g, 22.6 mmol), 2-hydroxyethyl acrylate (HEA, passed through neutral Al_2O_3 to remove stabilizer, 1.75 g, 15.1 mmol), 2-(dodecylthiocarbonothioylthio)-2-methylpropionic acid pentafluorophenyl ester (PFP-DMP, 0.2 g, 0.377 mmol), 2,2′-azobis(2-methylpropionitrile) (AIBN, 30.9 mg, 0.188 μmol) and TFA (3.46 mL, 45.2 mmol) were dissolved in DMF (48.2 mL) and then stirred for 5 min at room temperature. The whole mixture was then degassed for 30 min using nitrogen and then reacted for 18 h at 70 °C. Monomer and TFA ratios were adjusted to produce desired molar ratio of quinine monomer in PFP-poly(quinine-co-HEA). An aliquot of purified PFP-poly(quinine-co-HEA) was taken and dissolved in MeOD for NMR analysis.

To purify the PFP-poly(quinine-co-HEA), the mixture was reprecipitated into diethyl ester from methanol three times, yielding a yellow powder product (or yellow gel if not dried completely). The resulting product was dissolved in deionized water and then dialyzed against deionized water overnight to remove any remaining monomer and RAFT agent. Dialyzed product was dried on freeze dryer. An aliquot of purified polymer was taken for NMR analysis in MeOD and SEC analysis in DMF.

To synthesize DBCO-poly(quinine-co-HEA), dibenzocyclooctyne-amine (1.5 eq.) and PFP-poly(quinine-co-HEA) (1.0 eq.) were mixed a capped 20 mL glass vial using DMF (2 mL) as solvent at room temperature for 16 h. The crude product was purified by precipitated into diethyl ester from methanol for three times, yielding a dark yellow polymer product. The resulting product was dissolved and then dialyzed against water overnight to remove any remaining DBCO-amine. Dialyzed product was dried on freeze dryer. An aliquot of purified polymer was taken for NMR analysis in MeOD and SEC analysis in DMF.

Polyplexes formation

Polymers were dissolved in 0.0507 M acetic acid at concentration of 10 mg/mL and siRNA-Cy3 was dissolved in DNase/RNase-Free Water provided by supplier's kit at a stock concentration of 10 μM. The polymer solution and siRNA solution were mixed thoroughly by pipetting up and down in base culture media in the absence of any FBS or PSA and allow additional 30 min for polyplexes formation before adding to cells. Polyplexes were prepared at different N/P ratio but with constant final siRNA concentration of 50 nM. Polyplexes of polymer/plasmid were prepared by the same method, except that plasmid solution was dissolved in deionized water at storage concentration of 500 ng/mL and treated at final concentration of 3.33 ng/μL. The total volume of each well is 150 μL on 48-well plate and 900 μL on 35 mm confocal dish.

DNA encapsulation efficiency (gel electrophoresis)

To evaluate DNA condensation ability of DBCO-poly(quinine-co-HEA) and poly(quinine-co-HEA), gel electrophoresis and DNA dye exclusion assay was used to measure amount of free DNA after polyplexes formation. Polyplexes of different N/P ratio were prepared according to previous method and was loaded on Invitrogen™ E-Gel™ EX Agarose Gels. Gel image was captured after running for 10 min.

DNA encapsulation efficiency measurement (dye exclusion assay)

As for PicoGreen dye exclusion assay, Qubit™ dsDNA Quantification Assay Kit was used to measure accurate DNA encapsulation efficiency according to manufacturer's instructions. Briefly, polyplexes were prepared as previously described with different N:P ratio at the same dose of 1000 ng plasmid. PicoGreen dye working solution was then incubated with polyplexes or plasmid standard (0 to 2000 ng) for 2 min. Fluorescence was measured at 485/530 nm by plate reader. Specifically, quinine was also excited at dye excitation wavelength and its weak emission was slightly affecting fluorescence intensity of DNA binding dye. To elucidate quinine effects, bare polymer solution at the same polymer dose was performed as negative control and its weak emission was calculated as blank value for each N/P ratio.

Click reaction of azido-dye and DBCO-polymer/polyplexes

To evaluate DBCO groups level on polyplexes, a fluorescent dye, 3-azido-7-hydroxycoumarin (azido-coumarin), was used to react with DBCO group at 1: 1 molar ratio in 10 mM HEPES

buffer. Azido-coumarin will emit fluorescence at 477nm with excitation at 404nm after reacting with alkyne. DBCO-poly(quinine-co-HEA) was mixed with plasmid to perform polyplexes as previously described at polymer concentration of 2 mM. Azido-coumarin of 2 mM was then mixed with DBCO-polyplexes or DBCO-polymer at 1: 1 equal volume in HEPES buffer for 10 min. Fluorescence intensity was recorded by plate reader.

Gold/polyplexes preparation

We used a layer-by-layer strategy to make gold/polyplexes (Figure S2). Briefly, stock 30 nm gold nanoparticles were centrifuged at 15000 rpm for 10 min to remove stabilizer and resuspended in 0.2 mM HEPES (PH = 7.0) at final OD of 1. 11-mercaptoundecanoic acid (11-MUA) were dissolved in DMSO at 10 mg/mL as stock solution. 1 mL of gold nanoparticles were then mixed with 10 μL 11-MUA in 990 deionized water contains 10 μL NaOH (1 M). Mixtures were shaking at Eppendorf smart block for shaking at 2000 rpm for 30 min at room temperature and followed by centrifuge at 15000 rpm for 10 min to remove free 11-MUA.

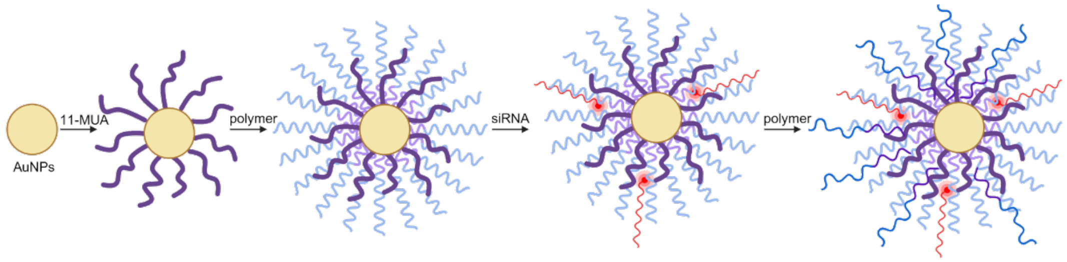

Figure S2. Illustration of Au/polyplexes fabrication. Particles were centrifuged to remove unconjugated 11-MUA and statically incubated with polymer and siRNA to form layer-by-layer coated Au/polyplexes nanoparticles.

To make gold/polyplexes for siRNA delivery on cells, the 11-MUA/Au nanoparticles were resuspended in sterile 10 mM HEPES at OD of 1 and then mixed with polymer solution in F-12K media without any supplements at different ratio. The mixture was incubated statically at room temperature for 30 min and followed by adding siRNA for another 30 min and ended up with adding polymer for 30 min. For siRNA delivery, final siCy3 dose is 10 nM, polymer dose is 133 μg/mL (half dose for each layer) and Au dose is 33, 17, 8, 4 x 10^{-3} OD.

Specifically, both resuspending and layer coating were performed in 0.2 mM HEPES buffer for DLS and zeta potential, and in water for TEM images.

In the following text, bare gold means commercial 30 nm gold nanoparticles and 11-MUA@AuNPs means 11-MUA conjugated gold nanoparticles. DBCO-poly(quinine-co-HEA)@AuNPs and poly(quinine-co-HEA)@AuNPs represents nanoparticles of 11-MUA@AuNPs/DBCO-poly(quinine-co-HEA)/siRNA/ DBCO-poly(quinine-co-HEA) and 11-MUA@AuNPs/ poly(quinine-co-HEA)/siRNA/ poly(quinine-co-HEA).

Plasmid preparation

Plasmid was transfected and amplified using *E. Coli* Top 10. The amplified plasmid was purified by using E.Z.N.A.® Endo-Free plasmid mini kit II according to manufacturer's instructions. The purified plasmid was characterized by gel electrophoresis with positive control of DNA ladder and standard plasmid template from company, and its dose was measured on IMPLEN NanoPhotometer®. Plasmid was dissolved in deionized water and stored at -20 °C before use.

Mammalian cell culture

A549 cells were grown in 75 cm^2 Nunc cell culture flasks (Thermo Fisher, Rugby, UK) with Ham's F-12K (Kaighn's) Medium (F-12K) (Gibco, Paisley, UK) supplemented with 10% non-USA origin fetal bovine serum (FBS) purchased from Sigma Aldrich (Dorset, UK), 100 units/mL penicillin, 100 µg/mL streptomycin, and 250 ng/mL amphotericin B (PSA) (HyClone, Cramlington, UK) in the presence of 5% CO_2 at 37 °C.

HeLa cells were grown in 75 cm^2 Nunc cell culture flasks (Thermo Fisher, Rugby, UK) with High glucose DMEM (Gibco, Paisley, UK) supplemented with 10% non-USA origin fetal bovine serum (FBS) purchased from Sigma Aldrich (Dorset, UK), 100 units/mL penicillin, 100 µg/mL streptomycin, 250 ng/mL amphotericin B (PSA) (HyClone, Cramlington, UK) and 1 mM sodium pyruvate in the presence of 5% CO_2 at 37 °C.

HeLa-GFP cells were grown in 75 cm^2 Nunc cell culture flasks (Thermo Fisher, Rugby, UK) with high glucose Dulbecco's Modified Eagle Medium (DMEM) (Gibco, Paisley, UK) supplemented with 10% non-USA origin fetal bovine serum (FBS) purchased from Sigma Aldrich (Dorset, UK), 100 units/mL penicillin, 100 µg/mL streptomycin, 250 ng/mL amphotericin B (PSA) (HyClone, Cramlington, UK), 1 mM sodium pyruvate and 2 mM L-glutamine in the presence of 5% CO_2 at 37 °C.

Jurkat cells were grown in 75 cm^2 Nunc cell culture flasks (Thermo Fisher, Rugby, UK) with

Roswell Park Memorial Institute (RPMI) 1640 Medium (Gibco, Paisley, UK) supplemented with 10% non-USA origin fetal bovine serum (FBS) purchased from Sigma Aldrich (Dorset, UK), 100 units/mL penicillin, 100 μg/mL streptomycin, and 250 ng/mL amphotericin B (PSA) (HyClone, Cramlington, UK) in the presence of 5% CO_2 at 37 ºC.

Both HeLa cells and HeLa-GFP cells are gifts from Steve Royle's group at Warwick Medical School. Specifically, HeLa-GFP cells were HeLa cells permanently transfected with plasmid of CLTA-FKBP-GFP, GFP tagged clathrin.

Gene delivery

Gene delivery is made up of two steps: metabolic glycan labelling and polymeric gene delivery. To label cell surface glycans with azido-groups, cells were pre-incubated with $Ac_4ManNAz$ for 72 h. $Ac_4ManNAz$ was dissolved in DMSO first before adding to cells. To minimize DMSO effects on cell viability of long-time incubation, $Ac_4ManNAz$ was dissolved at 40 mM stock concentration.

Briefly, A549, HeLa and HeLa-GFP cells were pre-seeded in 48-well plate at cell density of 5 × 10^4 per mL and then treated with 50 μM $Ac_4ManNAz$ (with final volume per well of 250 μL containing 0.1% DMSO) for 72 h before gene delivery. Cells were washed with cold DPBS for three times and then incubated with 150 μL polyplexes for 10 min (or 30 min, 1 H and 2 H for time-dependency siRNA-Cy3 delivery). Cells were then washed with cold DPBS for once and followed by full media culture for additional h. For siRNA-Cy3 delivery, cells were immediately trypsinized after 10 min delivery and then fixed using 4% formaldehyde before flow cytometry analysis under PE channel. For siRNA-GFP and GFP plasmid delivery, cells were washed and supplied with full media and cultured for an additional 12 h and 48 h, respectively, and then were fixed using formaldehyde before flow cytometry analysis under FITC channel.

For Jurkat cells, cells were pre-seeded in 10 cm^2 culture dishes at cell density of 1.25 × 10^5 per mL and incubated with 50 μM $Ac_4ManNAz$ (with final volume per dish of 10 mL containing 0.1% DMSO) for 72 h. On the day of gene delivery, cells were collected and washed with cold DPBS three times by centrifuging at 300 g for 5 min before gene delivery. Jurkat cells were counted using Countess II Automated Cell Counter and seeded into u-bottom 96-well plate at cell number of 150k per well with volume of 30 μL. Cells were then incubated with 45 μL

polyplexes for 10 min and then washed with cold DPBS for twice. For siRNA-Cy3 delivery, cells were fixed with formaldehyde immediately after delivery and used for flow cytometry analysis. For plasmid delivery, cells were supplied with 300 µL full media and cultured for additional 48 h. Cells were then fixed using formaldehyde and analyzed by BD Accuri™ C6 Plus Flow Cytometer.

Flow cytometry

Adherent cells, A549, HeLa and HeLa-GFP cells were trypsinized and incubated with 4% formaldehyde solution for 15 min at room temperature and then washed with cold DPBS for three times. Cells were resuspended in cold DPBS supplied with 10% FBS and stored on ice during flow cytometry analysis. Cells were filtered through 40 µm cell strainer before loading on flow cytometer. Flow cytometry data was analyzed using flow jo software.

Confocal microscopy

A549 cells were pre-seeded in 35 mm confocal dishes at cell density of 5×10^4 per mL and treated with 50 µM $Ac_4ManNAz$ (with final volume per dish at 1500 µL containing 0.1% DMSO) for 72 h. Cells were washed with cold DPBS for three times and then incubated with 900 µL polyplexes containing siRNA-Cy3 with final concentration of 50 nM for 10 min. Cells were washed with cold DPBS for once and then supplied with full media to culture for an additional 12 h. Cells were incubated with NucBlue™ Live ReadyProbes™ Reagent (Hoechst 33342) and LysoTracker™ Deep Red for 30 min at room temperature and then washed with cold DPBS for three times. Cells were fixed using 4% formaldehyde for 15 min and washed with cold DPBS for three times. Cells were covered with foil and stored in fridge and imaged within one week. To minimize fluorophore overlapping among organelle staining dyes and siRNA-Cy3, sequential confocal scanning mode was used. Specifically, nuclei were imaged under DAPI channel with excitation wavelength of 405 nm and emission wavelength of 430 – 470 nm; lysosomes were imaged under Alexa Fluor 640 nm with excitation wavelength of 640 nm and emission wavelength of 680 – 780 nm; siRNA-Cy3 was imaged under Cy3 channel with excitation wavelength of 561 nm and emission wavelength of 600 – 640 nm. DAPI was grouped with Alexa Fluor 640 and Cy3 was grouped with bright filed (using laser 405 nm), respectively. All images of cells with different treatment were captured under the same microscope setting. Images shown in manuscript were applied with the same increment of

brightness and contrast. Colocalization analysis of zoomed-in images were performed using JACoP in Fiji software and quantitative fluorescence intensity (integrated density) was calculated in Fiji software.

Resazurin assay

To assay toxicity of polymeric gene delivery, resazurin assay was used to measure metabolic activity of cells with different treatment. Briefly, A549, HeLa or HeLa-GFP cells were pre-seeded in 96-well plate at cell density of 2.5×10^4 per mL and treated with 50 µM $Ac_4ManNAz$ (with final volume per well of 100 µL containing 0.1% DMSO) for 72 h. Cells were washed with cold DPBS for three times and then treated with 75 µL polyplexes for 10 min. Cells were then washed with cold DPBS once and then replaced with fresh culture media to culture for additional 24 h before resazurin assay.

Table S1. Molar ratios of quinine monomer in polymer structure under different conditions.

polymer	$[M_{quinine}]$: $[M_{HEA}]$: [CTA] : [AIBN] : [TFA]	^{19}F signal	%Quinine in polymer (molarity)
PFP-poly(quinine-co-HEA)[a]	40 : 60 : 1 : 0.2 : N/A	N/A	14
PFP-poly(quinine-co-HEA)[a]	40 : 60 : 1 : 0.2 : 80	Yes	6.5
PFP-poly(quinine-co-HEA)[b]	40 : 60 : 1 : 0.2 : 80	Yes	7.5
PFP-poly(quinine-co-HEA)[a]	50 : 50 : 1 : 0.2 : 100	Yes	10.2
PFP-poly(quinine-co-HEA)[a]	60 : 40 : 1 : 0.2 : 100	yes	12

[a] degassing time of 30 min; [b] degassing time of overnight.

Table S2. SEC characterization of PFP-poly($quinine_{0.12}$-co-$HEA_{0.88}$) and DBCO-poly($quinine_{0.12}$-co-$HEA_{0.88}$).

Polymer	$[M_{quinine}]$: $[M_{HEA}]$: [CTA] : [AIBN] : [TFA]	$M_{w,theo}$ (g/mol)	$M_{w,sec}$ (g/mol)	$M_{n,sec}$ (g/mol)	$Đ_{n,sec}$ (g/mol)	DP
PFP-poly($quinine_{0.12}$-co-$HEA_{0.88}$)	60 : 40 : 1 : 0.2 : 120	24.4k	11.8k	6.4k	1.86	18
DBCO-poly($quinine_{0.12}$-co-$HEA_{0.88}$)	60 : 40 : 1 : 0.2 : 120	24.6k	12.6k	7.1k	1.78	19

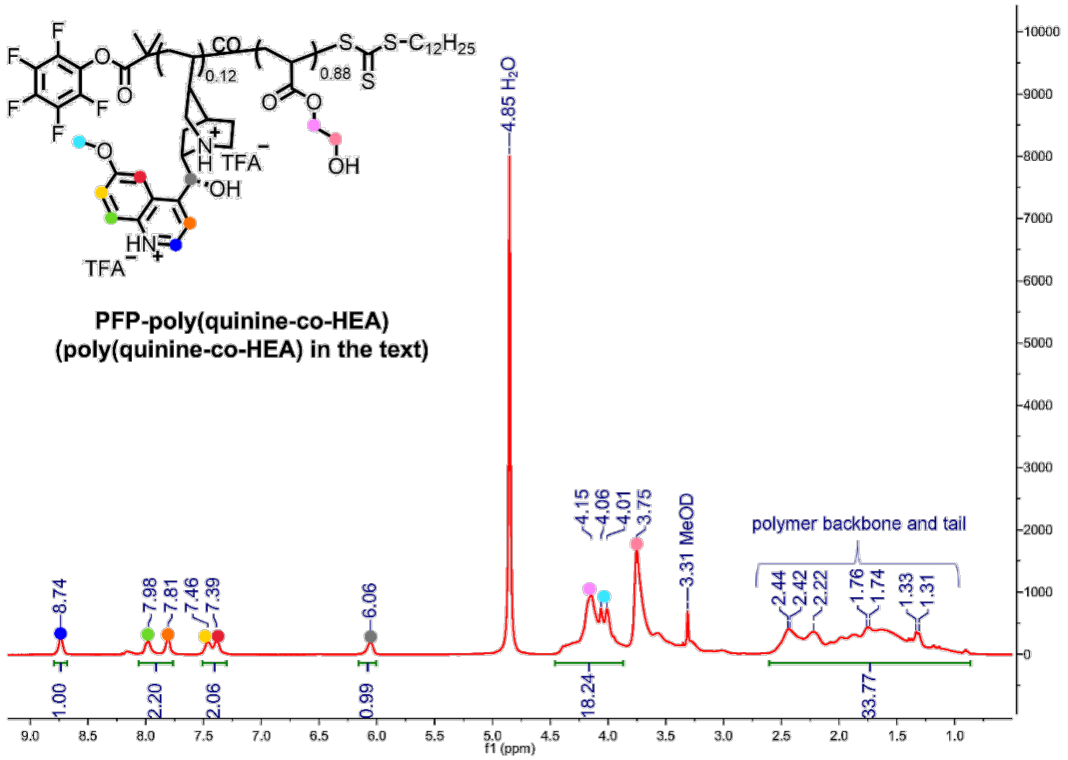

Figure S3. ^{1}H-NMR spectrum of PFP-poly(quinine$_{0.12}$-co-HEA$_{0.88}$). Polymers were dissolved in methonal-d$_4$ for ^{1}H-NMR on Bruker 400Hz. The Monomer ratio was calculated as previously reported.

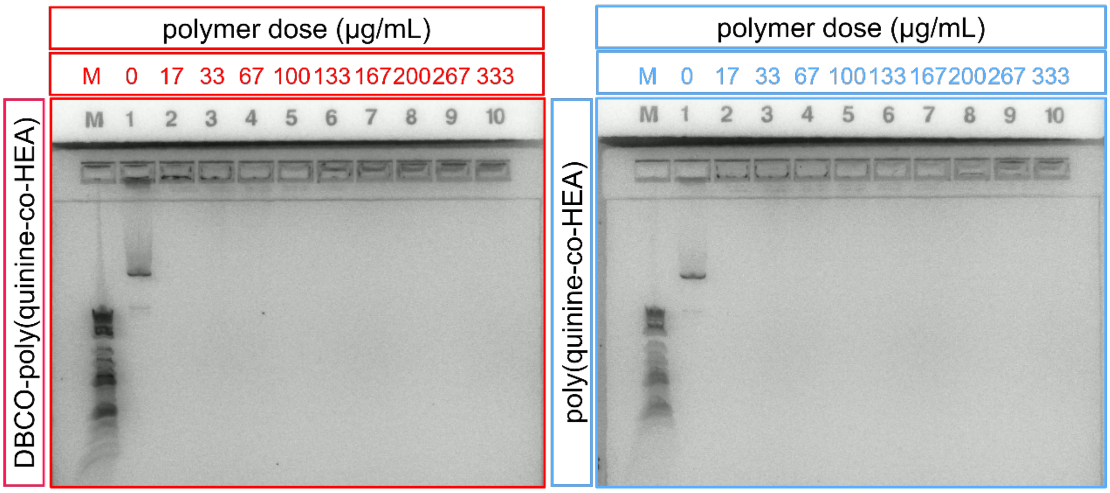

Figure S4. DNA agarose gel of DBCO-poly(quinine-co-HEA)/GFP-plasmid and poly(quinine-co-HEA)/GFP-plasmid polyplexes. Polyplexes were formulated at different N:P ratios. The loading DNA amount for each lane was 50 ng. Gel was run on E-Gel™ Power Snap Electrophoresis System using 1% pre-made EX agarose gel for 10 min and imaged immediately afterwards.

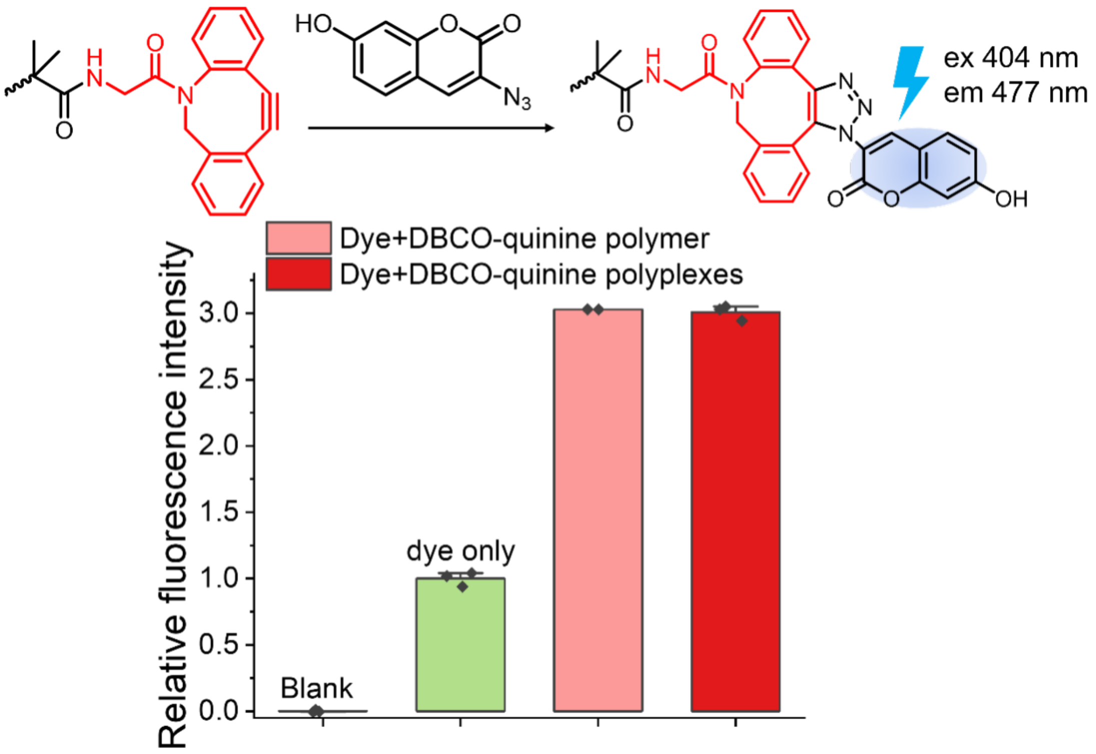

Figure S5. Evaluation of DBCO groups on DBCO-polyplexes. 3-azido-7-hydroxycoumarin (1 mM) was incubated with DBCO polymer or DBCO-polyplexes with the same polymer concentration of 1 mM in 10 mM HEPES buffer (pH = 7.0) and fluorescence of solution was recorded. Excitation wavelength: 404 nm; emission wavelength: 477 nm.

Table S3. Size and zeta potential characterization of DBCO-poly(quinine-co-HEA) and poly(quinine-co-HEA) under different N:P ratios. Poly(quinine$_{0.12}$-co-HEA$_{0.86}$) and DBCO-poly(quinine$_{0.12}$-co-HEA$_{0.86}$) were mixed with plasmid at different mass ratio in base media for 30 min before measurements.

Polymer	Polymer dose (μg/mL)	Hydrodynamic diameter (nm)	Zeta potential (mV)
DBCO-poly(quinine-co-HEA)	33	192.6 ± 16.4	7.1 ± 0.1
	67	150.9 ± 6.6	6.2 ± 0.2
	133	180.1 ± 17.7	5.7 ± 0.1
poly(quinine-co-HEA)	33	168.7 ± 8.5	7.0 ± 0.1
	67	128.3 ± 6.7	6.4 ± 0.1
	133	191.2 ± 19.3	5.3 ± 0.1

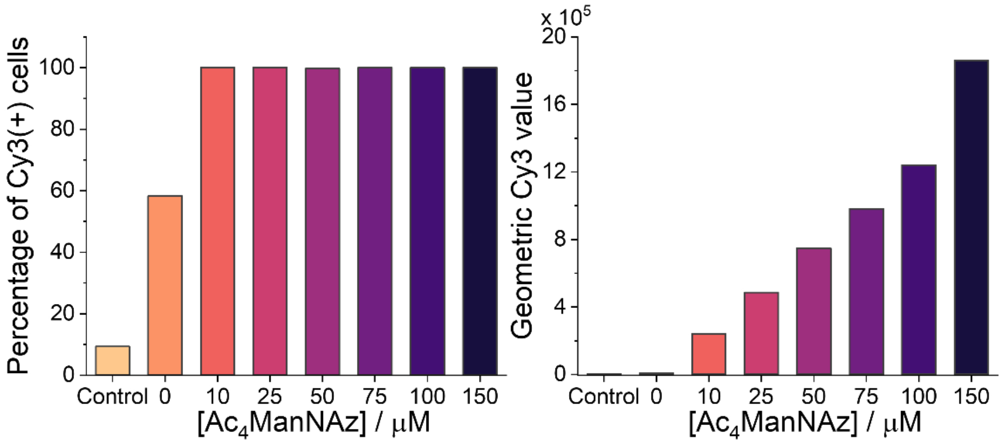

Figure S6. DBCO-Cy3 (50 μM) labelling on A549 cells under different dose of $Ac_4ManNAz$ pre-treatment of 72 h.

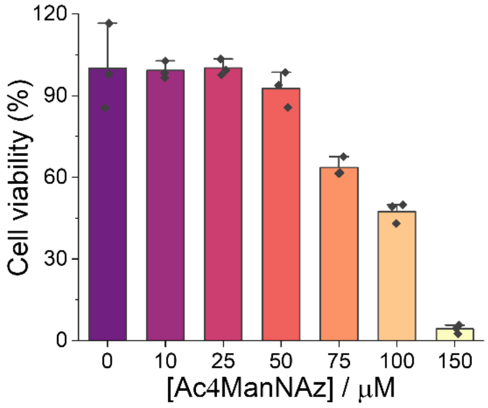

Figure S7. Cell viability of DBCO-Cy3 (50 μM) labelling on A549 cells under different dose of $Ac_4ManNAz$ pre-treatment of 72 h.

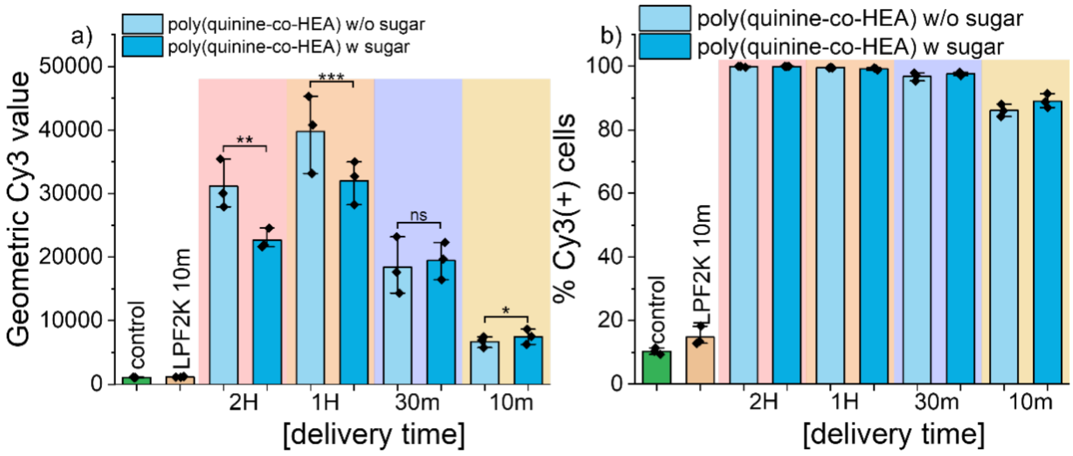

Figure S8. Time dependent delivery screening of siRNA-Cy3 by poly(quinine-co-HEA) into A549 cells with or without azido-sialic acid installation. a) Geometric mean Cy3 value of

cells; b) Percentage of Cy3 positive cells under different conditions. w-sugar = Ac_4ManAz labelled cells, w/o means without. N = 3 (Paired Sample t Test; ns: $p \geq 0.05$, $*p \leq 0.05$, $**p \leq 0.01$, $***p \leq 0.001$).

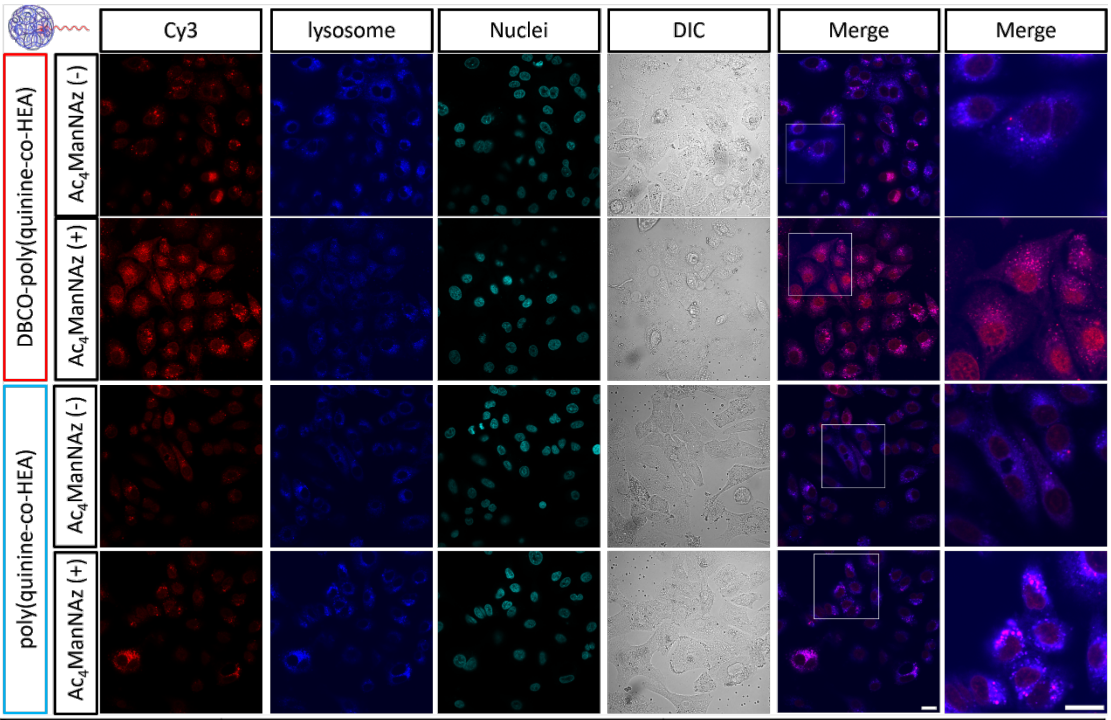

	DBCO-poly(quinine-co-HEA)		poly(quinine-co-HEA)	
	$Ac_4ManNAz$(-)	$Ac_4ManNAz$(+)	$Ac_4ManNAz$(-)	$Ac_4ManNAz$(+)
Pearson's coefficient	0.85	0.73	0.79	0.89
Overlap coefficient	0.97	0.89	0.95	0.97

Figure S9. Original confocal images of siRNA-Cy3 10 min-delivery by DBCO-poly(quinine-co-HEA) and poly(quinine-co-HEA) on A549 cells with or without 72 h 50 µM $Ac_4ManNAz$ pre-treatment. Colocalization analysis of zoomed-in images are performed using JACoP in Fiji software. Red: siRNA-Cy3; blue: lysosome; cyan blue: nuclei; grey: bright field. Scale bar: 20 µm.

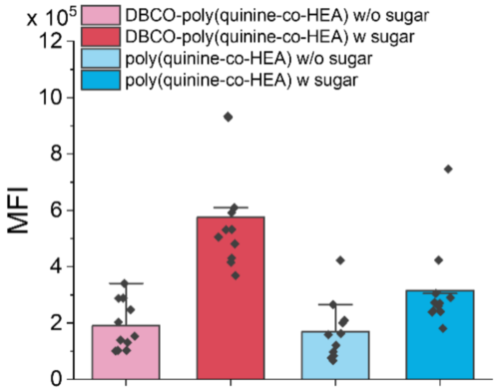

Figure S10. Quantitation of fluorescence intensity from confocal images, in Figure S9. A549 cells were treated with DBCO-poly(quinine-co-HEA)/siCy3 and poly(quinine-co-HEA)/siCy3 for 10 min following incubation for 72 h of 50 µM $Ac_4ManNAz$, or a negative control.

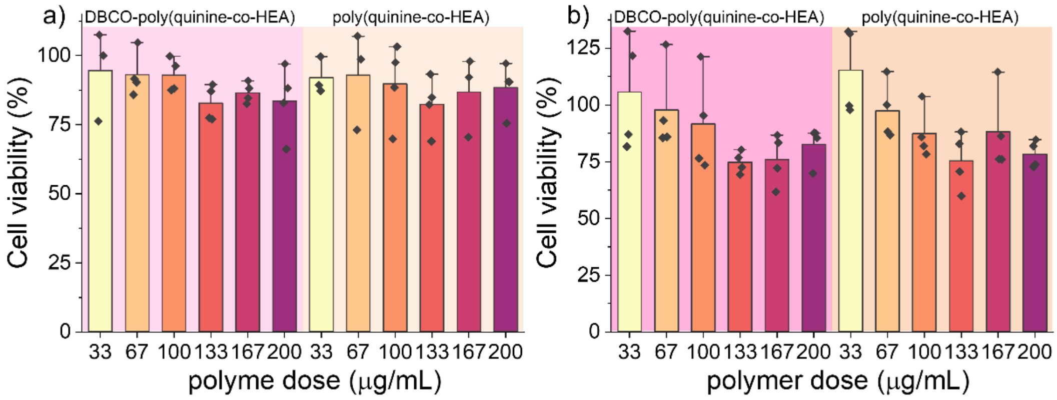

Figure S11. Cell viability of A549 cells with different dose of DBCO-poly(quinine-co-HEA) and poly(quinine-co-HEA) in the presence of 72 h pre-treatment of 50 µM Ac4ManNAz (a) or not (b). A549 cells were incubated with polymer for 10 min and then washed and cultured for an additional 24 h before resazurin assay.

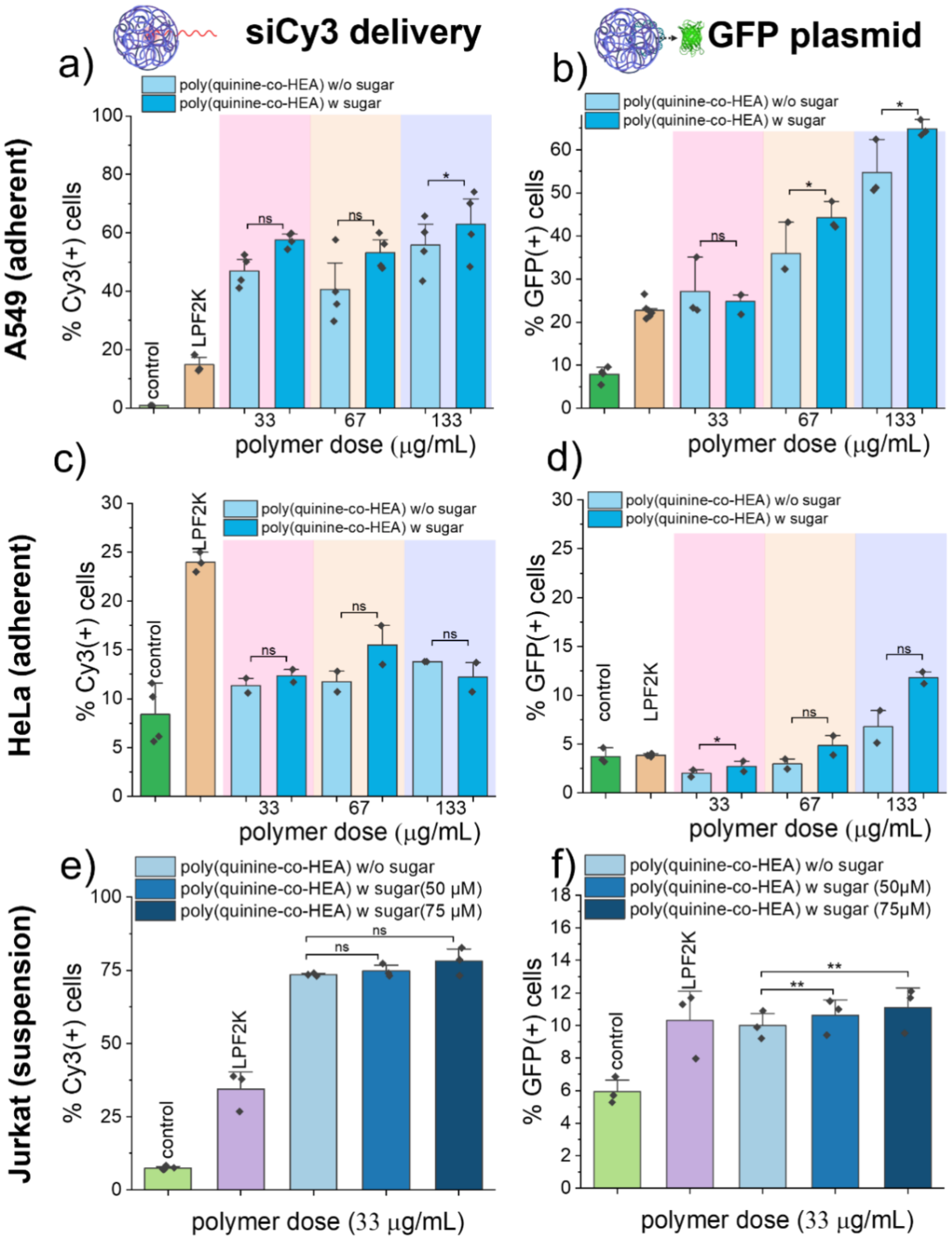

Figure S12. Dose dependency of siRNA-Cy3 and GFP plasmid delivery on three cell lines. A549 cells: a) siCy3 and b) GFP plasmid; HeLa cells: c) siCy3 and d) GFP plasmid; Jurkat cells: e) siCy3 and f) GFP plasmid. N=3 (Paired Sample t Test; ns: p ⩾ 0.05, *p ⩽ 0.05, **p ⩽ 0.01, ***p ⩽ 0.001).

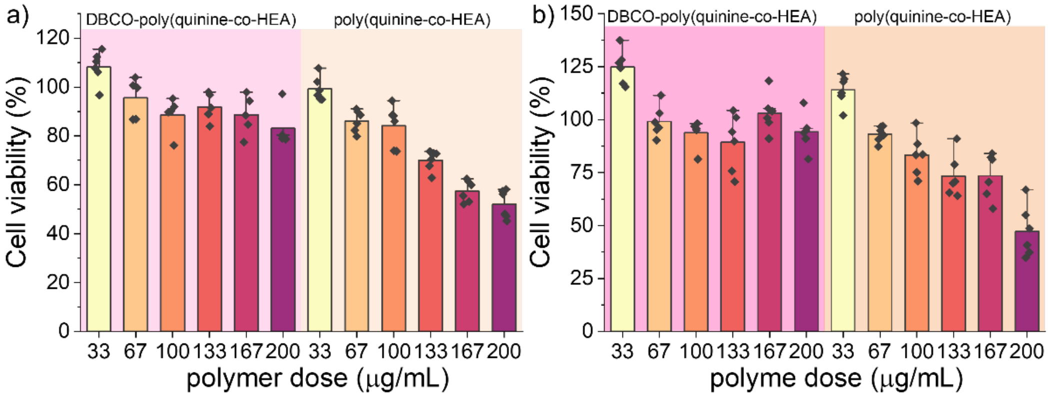

Figure S13. Cell viability of HeLa cells with different dose of DBCO-poly(quinine-co-HEA) and poly(quinine-co-HEA) in the presence of 72 h pre-treatment of 50 μM Ac4ManNAz (a) or not (b). HeLa cells were incubated with polymer for 10 min and then washed and cultured for an additional 24 h before resazurin assay.

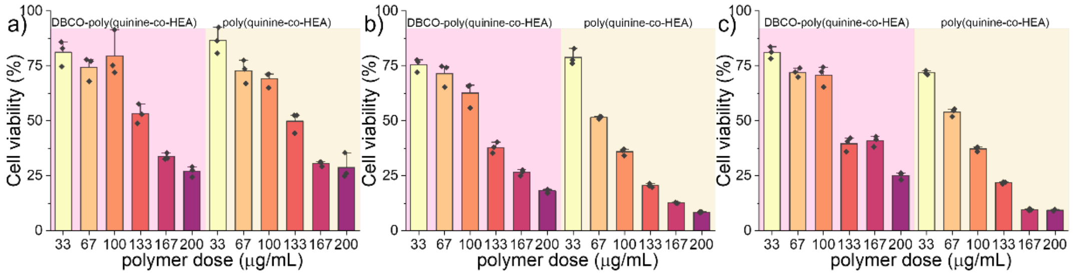

Figure S14. Cell viability of Jurkat cells with different dose of DBCO-poly(quinine-co-HEA) and poly(quinine-co-HEA) in the presence of 72 h pre-treatment of 0 μM (a), 50 μM (b) or 75 μM (c) Ac4ManNAz. Jurkat cells were incubated with polymer for 10 min and then washed and cultured for additional 24 h before resazurin assay.

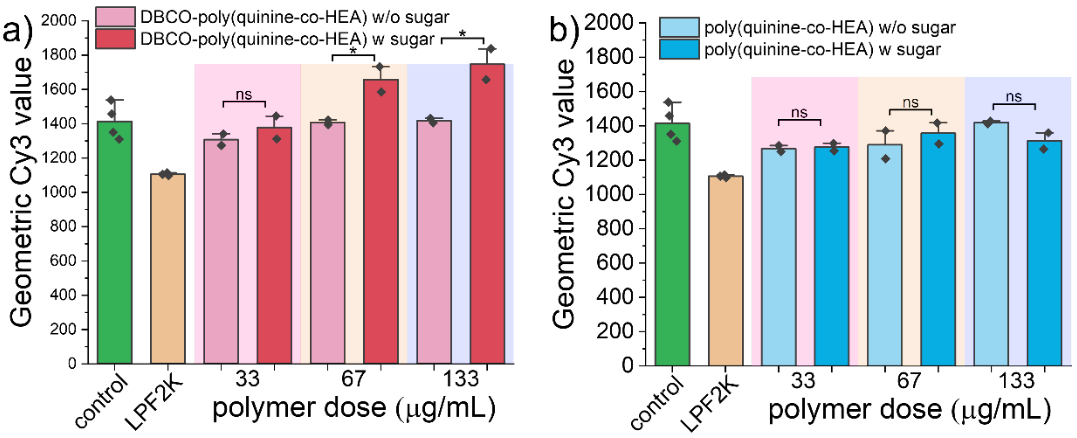

Figure S15. Dose dependency of siCy3 delivery on HeLa cells. Geometric Cy3 value of HeLa cells with 10 min delivery by a) DBCO-poly(quinine-co-HEA) and b) poly(quinine-co-HEA). N=3 (Paired Sample t Test; ns: p $\geqslant$ 0.05, *p $\leqslant$ 0.05, **p $\leqslant$ 0.01, ***p $\leqslant$ 0.001).

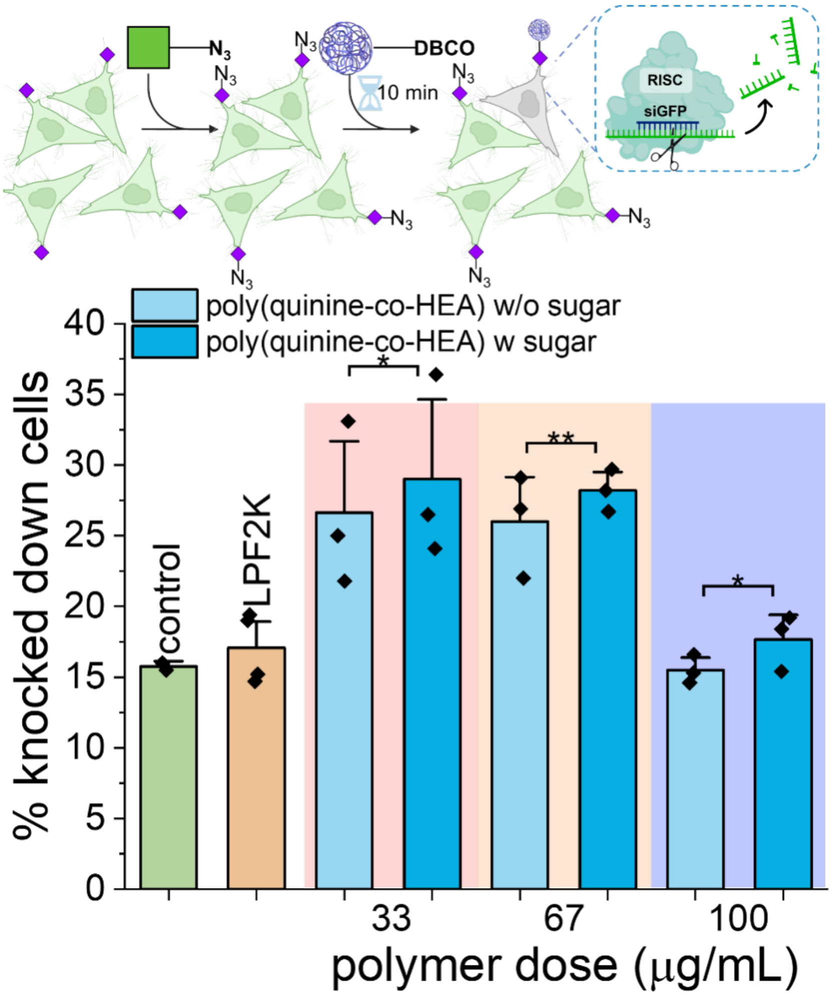

Figure S16. Dose dependency of siGFP delivery by poly(quinine-co-HEA) on HeLa-GFP cells with or without 72 h 50 μM $Ac_4ManNAz$ pre-treatment. N=3 (Paired Sample t Test; ns: p

⩾ 0.05, *p ⩽ 0.05, **p ⩽ 0.01, ***p ⩽ 0.001).

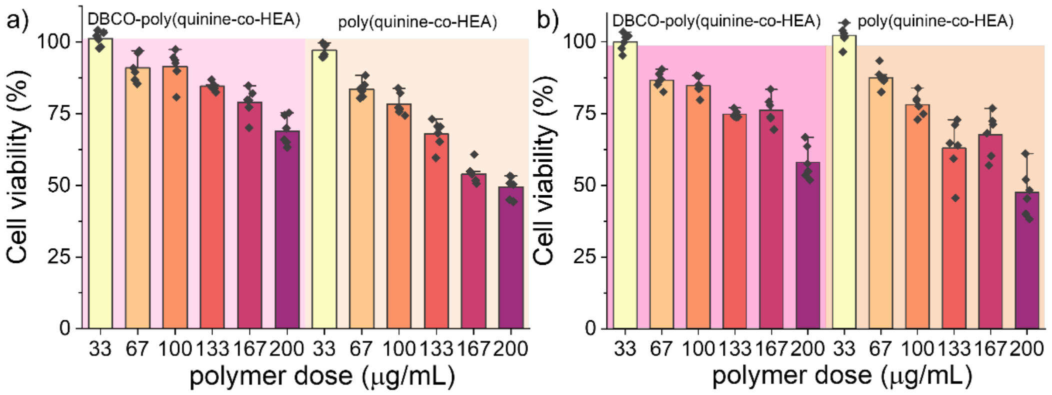

Figure S17. Cell viability of HeLa-GFP cells with different dose of DBCO-poly(quinine-co-HEA) and poly(quinine-co-HEA) in the presence of 72 h pre-treatment of 50 μM $Ac_4ManNAz$ (a) or not (b). HeLa-GFP cells were incubated with polymer for 10 min and then washed and cultured for an additional 24 h before resazurin assay.

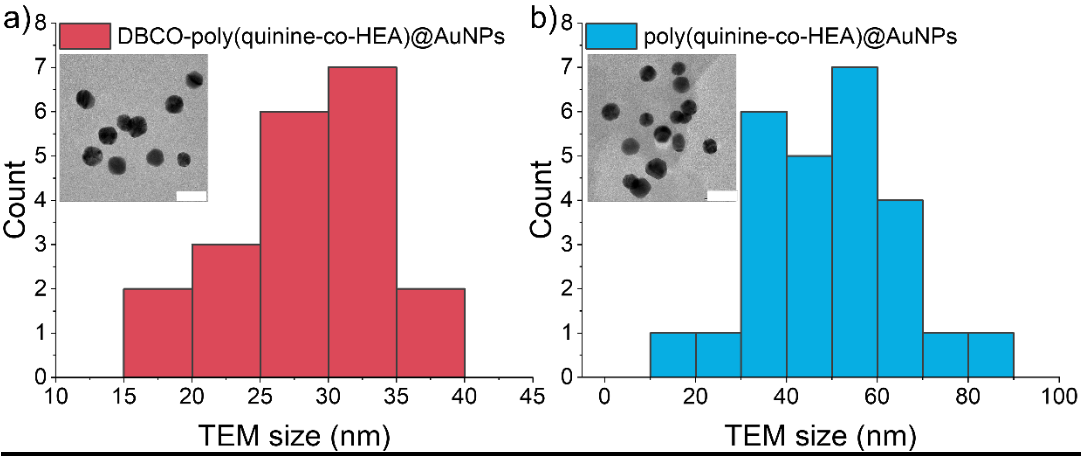

c) Particles	Au dose (OD × 10^{-3})	Hydrodynamic diameter (nm)	Zeta potential (mV)
Bare AuNPs	33	43.6 ± 0.4	-31.86 ± 8.9
11-MUA@AuNPs	33	43.8 ± 1.4	-32.39 ± 11.0
DBCO-poly(quinine-co-HEA)@AuNPs	33	164.3 ± 12.8	52.8 ± 1.3
poly(quinine-co-HEA)@AuNPs	33	168.0 ± 24.8	55.3 ± 2.3

Figure S18. Size and surface charge characterization on Au/DBCO-polyplexes and Au/polyplexes. Representative TEM of a) DBCO-poly(quinine-co-HEA)@AuNPs and b) poly(quinine-co-HEA)@AuNPs at final Au concentration of 33 × 10^{-3} OD, scale bar = 50 nm.

c). Dynamic light scattering and zeta potential characterizations of polymer coated particles.

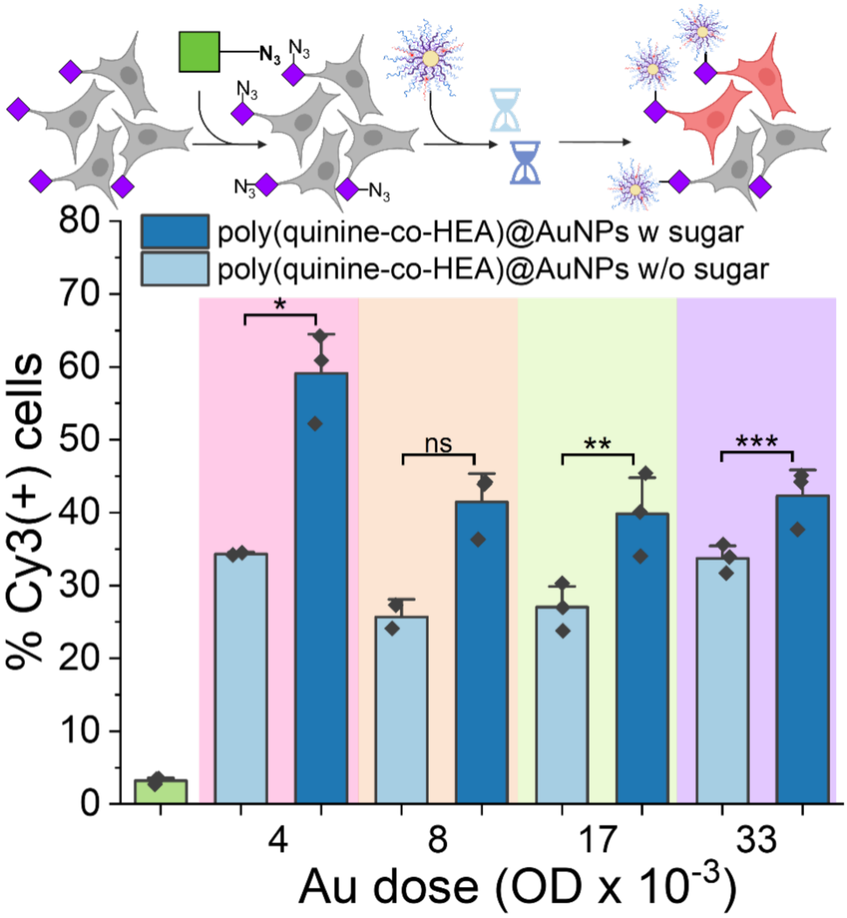

Figure S19. Dose dependency of siCy3 delivery by poly(quinine-co-HEA)@AuNPs nanoparticles on A549 cells with and without Ac_4ManNAz. Cells were incubated with poly(quinine-co-HEA)@AuNPs nanoparticles for 10 min and then washed with cold DPBS for three times before flow analysis. N=3 (Paired Sample t Test; ns: $p \geqslant 0.05$, *$p \leqslant 0.05$, **$p \leqslant 0.01$, ***$p \leqslant 0.001$).
